# The clinical pattern of renal diseases in the nephrology in-patient unit of the Yaounde General Hospital in Cameroon: a five-year audit

**DOI:** 10.11604/pamj.2015.21.205.5945

**Published:** 2015-07-20

**Authors:** Francois Folefack Kaze, Forbin Elias Ekokobe, Marie Patrice Halle, Hermine Fouda, Alain Patrick Menanga, Gloria Ashuntantang

**Affiliations:** 1 Department of Internal Medicine and Specialties, Faculty of Medicine and Biomedical Sciences & Yaounde General Hospital, University of Yaounde 1, Yaounde, Cameroon; 2 Department of Internal Medicine, Faculty of Medicine and Pharmaceutical Sciences & Douala General Hospital, University of Douala, Douala, Cameroon; 3 Department of Internal Medicine and Specialties, Faculty of Medicine and Biomedical Sciences & Douala Hospital, University of Yaounde 1, Yaoundé, Cameroon

**Keywords:** Kidney diseases, clinical pattern, Cameroon, Sub-Saharan Africa

## Abstract

**Introduction:**

Kidney diseases are a growing worldwide problem and one of the major public health threats. We analyzed the spectrum of kidney diseases seen over a five-year period in the nephrology in-patient unit of the Yaounde general hospital.

**Methods:**

This was a retrospective analysis of 225 medical records of patients admitted from January 2005 to December 2009 in the unit with a discharge diagnosis of kidney and urinary tract diseases. The first hospitalization was considered for patients admitted several times for the same disease. Socio-demographic and clinical patient data were recorded.

**Results:**

The patients mean age was 44.8±16 years with 135 (60%) males and 211 (93.8%) emergency admissions. All 139 (61.8%) patients with chronic kidney disease (CKD) had chronic renal failure. Acute kidney injury (AKI) (28%), nephrotic syndrome (7.6%), renal colic (1.3%) and acute pyelonephritis (1.3%) were other patterns observed. Chronic glomerulonephritis (25.9%), hypertension (22.3%) and diabetes (20.1%) were the main etiological factors of CKD. All AKI patients were in stage RIFLE-F. AKI was secondary to parenchymal (58.7%), functional (25.4%) and obstructive (15.9%) etiologies. Black water fever (36.4%), sepsis (22.7%), drugs (18.2%), eclampsia (13.6%) and herbal concoctions (9.1%) were the etiologies of acute tubular necrosis while enterocolitis (56.2%), heart failure (31.3%) and digestive hemorrhage (12.5%) were the etiologies of functional AKI.

**Conclusion:**

The clinical pattern of renal diseases is dominated by advanced CKD and AKI secondary to preventable causes. This study suggests a need for an array of actions including sensitization, continuous medical education and strengthening of the health system.

## Introduction

Kidney diseases are a growing worldwide problem and constitute one of the major public health threats [1]. Whether acute or chronic, the progressive increase in prevalence is related to modifications in life style, an increase in life span, medical progress and nephrotoxin abuse [2]. Chronic kidney disease (CKD) is the leading kidney disease which causes end-stage renal disease (ESRD) and cardiovascular deaths. In developed countries, CKD is related to the high prevalence of diabetes and hypertension [3] while renal hypoperfusion, sepsis and nephrotoxin constitute the main etiologies of acute kidney injury (AKI) [4–7]. However, sub-Saharan African (SSA) countries face the dual burden of non-communicable and transmissible diseases which may predispose to an increase in prevalence and a modified spectrum of kidney diseases [8]. Despite epidemiological transition in this setting, CKD remains the leading kidney disease, affecting predominantly young male adults, in their economically productive years, with chronic glomerulonephritis, hypertension and diabetes as the main etiological factors [9–16] while AKI is mainly secondary to diarrhea, obstetric complications, toxins and infections [17, 18]. Few countries in SSA have nephrologists and where available, nephrology units are under-equipped and understaffed; hence, patients with kidney diseases are generally managed in general internal medicine wards [11]. In Cameroon at the time of conducting this study, only five nephrologists, working in the two main cities of Douala and Yaounde provided care for an estimated population of 20 million inhabitants [19]. Consequently, both community and hospital-based data on the types of kidney disease in the country are lacking. This study was designed to analyze the spectrum of kidney diseases seen over a five-year period in the main nephrology in-patient unit of the country in order to assess the disease burden and to design preventive measures.

## Methods

### Study setting

This was a retrospective analysis of medical records of patients admitted from January 2005 to December 2009 in the nephrology-cardiology-psychiatry unit of the Yaoundé General Hospital (YGH); the main tertiary hospital in the Cameroon capital city. This unit of 25 beds is part of the internal medicine department and constitutes the main national referral nephrology unit. The staff comprised three nephrologists taking care of patients mainly from the center region and neighborhood but also from other parts of the country. This study received administrative authorization from the YGH and was approved by the Ethic Committee of the Faculty of Medicine and Biomedical Sciences (FMSB) of Yaoundé 1 University, Cameroon.

### Data collection

During the study period, we used the in-patient register of the unit to identify all patients with a discharge diagnosis of diseases of the kidney and urinary tract. This list was then used to retrieve the individual medical records from the hospital archives department. We considered only the first hospitalization for patients admitted several times for the same disease. For those with several diagnoses, each diagnosis was counted separately. We excluded files with missing data and patients hospitalized for hemodialysis-related complications. Patient information recorded included age, gender, type of admission (emergency, elective), kidney and urinary tract disorder, comorbidities, type and etiological factors of AKI, as well as stage and etiological factors of CKD.

### Definitions

Acute kidney disease (AKD) referred to any kidney disease of less than three months duration including AKI. AKI was defined as sudden loss of kidney function with recovering within three months. AKI was then classified into functional, parenchymal and obstructive. The RIFLE classification was used to stage AKI severity [20]. CKD referred to any kidney disease of more than three months duration including chronic renal failure which corresponds to persistent glomerular filtration rate (GFR) [21]. Nephrotic syndrome was defined by the association of proteinuria >3.5g/24 hours, hypoalbuminemia < 3.0g/dl and edema. Chronic glomerulonephritis referred to a chronic renal failure in patient of younger age (< 45 years) with hypertension, proteinuria, haematuria and small size kidney on ultrasonography. Hypertension was defined as a systolic blood pressure (SBP) ≥140 mmHg and/or a diastolic blood pressure (DBP) ≥90 mmHg or use of blood pressure lowering medications. Diabetes mellitus was defined as fasting capillary glucose? 126 mg/dl or use of glucose control agents. All diagnoses were made by a nephrologist.

### Statistical analysis

Statistical analysis were performed using the SPSS^®^ 9 software for Windows (SPSS, Chicago, IL, USA). We have reported results as mean (standard deviation) and count (percentages).

## Results

### General profile of the study population

During the study period, 358 patients were hospitalized for kidney and urinary tract disorder among which 256 (71.5%) fulfilled the study criteria. Only 225 (87.9%) files were available for analysis divided into 139 (61.8%) CKD and 86 (38.2%) AKD. As presented in Table 1, the patients’ age ranged from 13 to 84 years with a mean of 44.8±16 years, 135 (60%) were males and 211 (93.8%) were emergency admissions. Hypertension was the main comorbidity recorded in 142 (63.1%) patients whereas 26 (11.6%) patients had HIV infection.

**Table 1:** Characteristics overall and by diagnostic categories

Characteristics	Overall n (%)	CRF n (%)	AKI n (%)	NS n (%)	RC n (%)	AP n (%)
n	225 (100)	139 (61.8)	63 (28.0)	17 (7.6)	3 (1.3)	3 (1.3)
Male sex	135 (60.0)	85 (61.2)	37 (58.7)	10 (58.8)	2 (66.7)	1 (33.3)
Emergency admission	211 (93.8)	139 (100)	63 (100)	3 (17.6)	3 (100)	3 (100)
Diabetes	56 (24.9)	49 (32.2)	5 (7.9)	1 (5.9)	0 (0.0)	1 (33.3)
Hypertension	142 (63.1)	113 (81.3)	24 (38.1)	2 (11.8)	2 (66.7)	1 (33.3)
Smoking	36 (16.0)	21 (15.1)	14 (22.2)	1 (5.9)	0 (0.0)	0 (0.0)
HIV infection	26 (11.6)	14 (10.1)	10 (15.9)	1 (5.9)	0 (0.0)	1 (33.3)

CRF – Chronic renal failure; AKI – Acute kidney injury; NS – Nephrotic syndrome; RC – Renal colic; AP – Acute pyelonephritis

#### Stage and etiological factors of CKD

**Figure 1:**
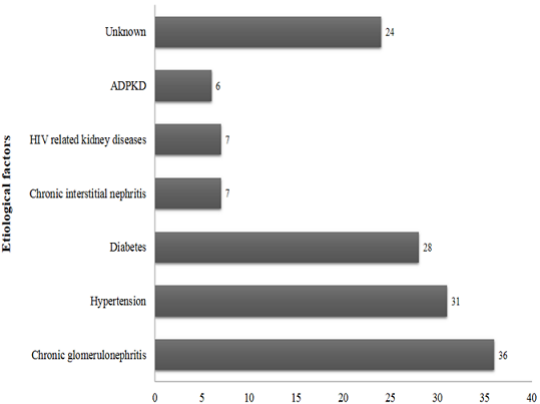
Type and frequency of etiological factors of chronic renal failure

All CKD patients had chronic renal failure. According to KDOQI classification, 4 (2.9%), 19 (13.7%) and 116 (83.4%) patients were respectively classified in stage 3, 4 and 5. Chronic glomerulonephritis (25.9%), hypertension (22.3%) and diabetes (20.1%) were the main etiological factors of CKD as presented in Figure 1.

### Type and etiological factors of AKI

**Figure 2:**
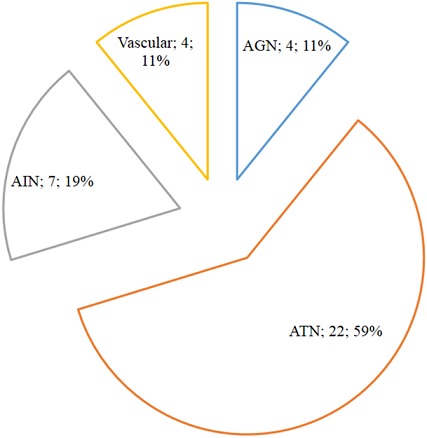
Etiological factors of acute kidney injury

All AKI patients were in RIFLE-F; with the suspected mechanism of injury being parenchymal, functional and obstructive in 37 (58.7%), 16 (25.4%) and 10 (15.9%) patients respectively. As presented in Figure 2, acute tubular necrosis (ATN) was the main suspected lesion in parenchymal AKI. Black water fever (n=8, 36.4%), sepsis (n=5, 22.7%), drugs (n=4, 18.2%), eclampsia (n=3, 13.6%) and herbal concoctions (n=2, 9.1%) were the etiologies of ATN. Acute interstitial nephritis was drug-induced (n=4, 57.1%), infectious (n=2, 28.6%) and due to lymphoma (n=1, 14.3%). Malignant nephroangiosclerosis (N=2, 50%) and ANCA-vasculitis (n=2, 50%) were the causes of vascular AKI whereas acute glomerulonephritis was exclusively post-infectious. Enterocolitis (n=9, 56.2%), heart failure (n=5, 31.3%) and digestive hemorrhage (n=2, 12.5%) were the etiologies of functional AKI meanwhile obstructive AKI was secondary to prostatic hypertrophy (n=6, 60%), nephrolithiasis (n=2, 20%) and cervical cancer (n=2, 20%).

### Etiology of nephrotic syndrome

Among 17 patients with kidney biopsy for nephrotic syndrome, 10 (58.8%) cases were identified as primary including focal and segmental glomerulosclerosis (n=8, 80%) and minimal change disease (n=2, 20%) lesions on kidney biopsy. The remaining cases were identified secondary to systemic lupus erythematosus (n=2, 28.6%), HIV-associated nephropathy (n=2, 28.6%), hepatitis B virus (n=1, 14.3%), filariasis (n=1, 14.3%) and non-steroidal anti-inflammatory drugs (n=1, 14.3%).

## Discussion

In a context marked by a scarcity of kidney specialists and units dedicated to renal care, we found that the clinical spectrum of diseases of the kidney and urinary tract observed in an in-patient medical ward were diverse and included CKD, AKI, nephrotic syndrome, renal colic and acute pyelonephritis. They affect mostly young males who frequently are admitted as emergency. This study revealed that kidney diseases dominated by CRF affect young individuals in their economically productive years and who are usually diagnosed late already in end stage as previously reported in similar settings; thus contrasting with the advanced age of patients in developed countries [3, 9–16]. The socio-cultural and economic factors favoring males in this setting could explain this gender bias in addition to being a risk factor for kidney disease [10–13, 16]. Chronic kidney disease was the main presentation accounting for nearly two-thirds of renal admissions with etiological factors similar to earlier reports [9, 10, 12–16]. The high prevalence of CKD reported in this setting could be related to the epidemiological transition with the increased prevalence of diabetes and hypertension coupled with unresolved transmissible diseases dominated by HIV/AIDS [8, 10]. In the present study, over half of the patients with HIV infection had CRF. We had previously reported a significant decrease in renal function as well as a high frequency of urine abnormalities in treatment-naïve HIV infected patients in this setting [22]. More than 8 in 10 patients with CKD were in ESRD, most often in a state of uremia requiring emergency renal replacement therapy; which probably reflects the late referral previously reported in this SSA setting [10, 16]. This could be explained by the under-resourced health systems and highlight the need for community-based awareness campaigns and continuous medical education for care-providers on the prevention and control of risk factors for CKD.

Acute kidney injury was the second most frequent presentation of kidney disease in our setting with unsurprisingly all patients in RIFLE-F. AKI was mainly from renal parenchymal disease dominated by acute tubular necrosis with etiologies similar to previous reports in developing countries [17, 18]. The high frequency of acute tubular necrosis likely reflects the longstanding evolution and inadequate management of functional AKI in other health facilities before transfer to our unit; the unique specialized unit with renal replacement therapy facilities in the city during the study period. As reported in other SSA settings, we found that malaria and its related disorders, pregnancy related complications and nephrotoxic drugs were the main etiologies of AKI implying more sensitization of populations on AKI prevention and health care-providers on early diagnostic and referral for better management. However, this etiological profile of AKI is quite different from developed countries where it usually occurs following cardiovascular procedures and sepsis in elderly persons [4–7]. The small number of patients with the nephrotic syndrome in this study reflects the local practice whereby only nephrotic patients with life-threatening complications are admitted to hospital. However, apart from diabetes, the potential secondary causes of nephrotic syndrome observed were dominated by endemic infections similar to those reported in SSA [23]. The absence of hypertension, as an entity in the disease spectrum observed in this study, results from the fact that in Cameroon, hypertension is managed by cardiologists except when it occurs in association with renal diseases. This study has some limitations. We excluded some patients due to incomplete files pointing out the weakness of archives service in our setting. The etiological diagnosis of renal disorders was mainly clinical due to the unavailability and unaffordability of renal histology as well as the late presentation of patients with CKD which reduces the sensitivity and specificity of renal histology. Moreover, most patients are self-funded, such that only patients who can pay for their care are admitted into hospital thus constituting a selection bias. Nevertheless, our study provides an overview of the spectrum of kidney diseases in our setting since it was conducted in the main national referral nephrology unit.

## Conclusion

This study revealed that patients admitted in a specialized nephrology unit for kidney and urinary tract diseases in Cameroon are mainly young adults in their economically productive years. The clinical pattern of disease is dominated by advanced CKD and AKI secondary to preventable causes. This study suggests a need for an array of actions including sensitization, continuous medical education and strengthening of the health system to improve the management of patients with kidney disease, and the implementation of a national program to prevent and control risk factors for kidney diseases.
